# Skin Regenerative Potential of Cupuaçu Seed Extract (*Theobroma grandiflorum*), a Native Fruit from the Amazon: Development of a Topical Formulation Based on Chitosan-Coated Nanocapsules

**DOI:** 10.3390/pharmaceutics14010207

**Published:** 2022-01-16

**Authors:** Geisa Nascimento Barbalho, Breno Noronha Matos, Gabriel Ferreira da Silva Brito, Thamires da Cunha Miranda, Thuany Alencar-Silva, Fernando Fabriz Sodré, Guilherme Martins Gelfuso, Marcilio Cunha-Filho, Juliana Lott Carvalho, Joyce Kelly do Rosário da Silva, Taís Gratieri

**Affiliations:** 1 Laboratory of Food, Drugs and Cosmetics (LTMAC), University of Brasilia, Brasilia 70910-900, DF, Brazil; geisabarbalho@gmail.com (G.N.B.); brenomatos15@hotmail.com (B.N.M.); thamiiresmiiranda@gmail.com (T.d.C.M.); gmgelfuso@unb.br (G.M.G.); marciliofarm@hotmail.com (M.C.-F.); 2 Laboratory of Automation, Chemometrics and Environmental Chemistry, University of Brasilia, Brasilia 70910-900, DF, Brazil; gabriel.brito@unb.br (G.F.d.S.B.); ffsodre@unb.br (F.F.S.); 3 Genomic Sciences and Biotechnology Program, Catholic University of Brasilia, Brasilia 70790-160, DF, Brazil; thuanyalencar@gmail.com (T.A.-S.); julianalott@gmail.com (J.L.C.); 4 Faculty of Medicine, University of Brasilia, Brasilia 70910-900, DF, Brazil; 5 Institute of Biological Sciences, Federal University of Pará, Belém 66075-110, PA, Brazil; joycekellys@yahoo.com.br

**Keywords:** nanocapsules, Cupuaçu seed extract, skin burn, skin regeneration, oleic acid

## Abstract

Scarless skin regeneration is a challenge in regenerative medicine. Herein, we explore the regenerative potential of a Cupuaçu seed extract (*Theobroma grandiflorum*) to develop an innovative skin regeneration formulation based on chitosan-coated nanocapsules. Cupuaçu seed extract significantly stimulated cell proliferation and migration. A reparative gene expression profile could be verified following extract treatment, which included high levels of MKI67, a cellular proliferation marker, and extracellular matrix genes, such as ELN and HAS2, which code for elastin and hyaluronic acid synthase 2. Formulations with Cupuaçu seed extract successfully entrapped into nanocapsules (EE% > 94%) were developed. Uncoated or coated nanocapsules with low-molecular-weight chitosan presented unimodal size distribution with hydrodynamic diameters of 278.3 ± 5.0 nm (PDI = 0.18 ± 0.02) and 337.2 ± 2.1 nm (PDI = 0.27 ± 0.01), respectively. Both nanosystems were physically stable for at least 120 days and showed to be non-irritating to reconstructed human epidermis. Chitosan coating promoted active penetration into undamaged skin areas, which were still covered by the stratum corneum. In conclusion, the present study demonstrated for the first time the biotechnological potential of the frequently discarded Cupuaçu seed as a valuable pharmaceutical ingredient to be used in regenerative skin products.

## 1. Introduction

Cupuaçu (*Theobroma grandiflorum* (Willd. ex Spreng.) K. Schum) presents a taxonomic relationship with cocoa (*Theobroma cacao* L.) and is one the most popular native fruits cultivated in the Brazilian Amazon region. The fruit pulp is used in a wide variety of food products, holding considerable economic importance for the Brazilian state of Pará, the largest producer and exporter [1]. The fruit’s almond, i.e., the Cupuaçu seed, represents about 30–45% of the fruit’s weight and is mainly discarded as industrial waste [2]. However, the seeds are highly rich in proteins, minerals, bioactive compounds, and fat, presenting a varied content of fatty acids (±60% dry weight) with characteristics and potential to be applied in cosmetics and pharmaceutical products [3]. Despite few pieces of research attempting to employ the fat from the Cupuaçu seeds in other food products as a substitute for cocoa butter [4], investigations on the biotechnological and therapeutic potentialities of this exotic fruit and its extracts have been limited to applications related to their antioxidant properties [5,6].

Fatty acids are essential metabolites with crucial cellular functions, including cell migration, development, differentiation, and proliferation [7], also influencing human cell processes. Unsaturated fatty acids, such as linoleic acids, are crucial arachidonic acid precursors, an essential inflammatory mediator in wound healing. Previous studies have suggested a relevant role and potential therapeutic application of fatty acids for skin wound healing [8,9]. They can modulate the release of pro-inflammatory cytokines, affecting neutrophil migration, increasing the wound healing tissue mass while decreasing the thickness of the necrotic cell layer edge around the wound, and ultimately modulating the closure of skin wounds [9]. Oleic acid, for instance, is an important constituent of Cupuaçu seeds and has been demonstrated to induce a faster wound closure when compared to linolenic and linoleic acids [10]. Even an oral supplementation of fatty acids has been demonstrated to accelerate the inflammatory phase of wound healing [11]. This evidence makes it possible to expect the Cupuaçu seed extract to hold therapeutic potential in speeding up the wound healing process.

Still, one of the most significant challenges in developing novel phytotherapeutic formulations is, after bioprospection and the establishment of the potential action, to deliver such extract effectively and safely to the target tissue.

Nanoencapsulation holds essential advantages for topical cutaneous application [12,13,14]. Depending on the nanosystem characteristic and coating material, nanoencapsulation can promote tissue interaction and active penetration, especially for highly lipophilic compounds [14,15,16], which generally show a low penetration into the inner skin layers. In this way, bioavailability and, consequently, formulation effectiveness can be increased.

Chitosan is a biodegradable and biocompatible polysaccharide based on glucosamine and N-acetylglucosamine used in wound healing formulations for presenting a moderate antimicrobial activity and a positive influence on the re-epithelialization and regeneration of the granular layer of wounds [17]. Chitosan also presents bioadhesive properties [18]. Therefore, when used as a nanosystem coating material, chitosan may stimulate the interaction of the nanosystem to the wounded tissue, enhancing formulation residence time in the application area and allowing for intimate contact between encapsulated compounds and the target site [15,19]. Chitosan has also been widely employed as a penetration enhancer for transient widening of the tight junctions between cells and increasing paracellular and intracellular penetration pathways [18,20], which stimulate the cutaneous absorption of drugs that would hardly cross the stratum corneum.

Therefore, this study aimed first at evaluating the stimulatory effect of Cupuaçu extract on human dermal fibroblast cultures and second at developing a safe and effective formulation to incorporate and deliver such extract to the skin. In addition, nanosystem chitosan coating potential advantages were evaluated in terms of formulation safety and marker penetration both into intact and injured skin models in vitro.

## 2. Materials and Methods

### 2.1. Material

Oleic acid (>95%), marginal acid (>95%), sodium methoxide, Span 60, polycaprolactone polymer, low-molecular-weight chitosan (MW 50–190 kDa, deacetylation = 75%), Vivaspin (MW = 100,000 Da, Sartorio), dimethyl sulfoxide (DMSO), and mucin (type II) were all purchased from Sigma-Aldrich (Steinheim, Germany). Ethyl ether, acetic acid, ethanol, and monobasic and dibasic sodium phosphate were acquired from Vetec (Rio de Janeiro, Brazil). The solvents methanol and n-hexane 95% were supplied by J.T. Baker (Philipsburg, PA, USA). Dulbecco’s Modified Eagle Medium (DMEM) was obtained from Thermo–Fisher Scientific (Waltham, MA, USA), and fetal bovine serum (FBS) was acquired from Gibco (Grand Island, NE, USA). Soy lecithin was purchased from Lipoid (Ludwigshafen, Germany). Polysorbate 80 was acquired from Merck (Darmstadt, Germany), whereas acetone and sodium chloride were from Serva (Rio de Janeiro, Brazil). Sodium hydroxide was purchased from Contemporânea Chemical Dynamics (São Paulo, Brazil). All analyses were performed using ultrapure water provided by Millipore (llkirkirch-Graffenstaden, France). Formvar resin and uranyl acetate were used for nanocapsules morphological analysis were purchased from Electron Microscopy Science (EMS, Hatfield, PA, USA). The reconstructed human epidermis test method kit was donated by EpiSkin™ (Rio de Janeiro, Brazil). The skin used in permeation analyses was obtained from porcine ears that had been generously provided by Bonasa (Brasilia, Brazil) and stored at −4 °C.

### 2.2. Obtainment and Characterization of Cupuaçu Seed Extracts

Cupuaçu seeds were dried in an oven at 40 °C for 24 h, which was the lowest temperature capable of reducing seeds humidity to lower than 20% in a relatively short time. Then, the seeds were ground, and fatty acids were extracted by a Soxhlet using hexane as a solvent for 3 h. The sample was analyzed in a GC-MS-QP2010 Ultra system (Shimadzu Corporation, Tokyo, Japan), equipped with an auto-injector (AOC-20i). The parameters of analysis were: silica capillary column Rxi-5ms (30 m × 0.25 mm; 0.25 μm film thickness) (Restek Corporation, Bellefonte, PA, USA); injector temperature: 250 °C; oven temperature programming: 60–240 °C (3 °C/min); helium as a carrier gas, adjusted to a linear velocity of 36.5 cm/s (1.0 mL/min); splitless mode injection of 1 μL of sample (oil 5 μL: hexane 500 μL); ionization by electronic impact at 70 eV; ionization source and transfer line temperatures at 200 and 250 °C, respectively. The mass spectra were obtained by automatically scanning every 0.3 s, with mass fragments in the range of 35–400 *m*/*z*. The components of the extract were identified by comparing their retention indices and mass spectra (molecular mass and fragmentation pattern) with data stored in the NIST [21].

### 2.3. In Vitro Biological Analyses

For cell viability, proliferation, and migration tests, fibroblasts used in this study were isolated from the dermis of healthy donors. They were produced as pilot batches that had been kindly provided by CellSeq Solutions (Belo Horizonte, Brazil), and cultured using Dulbecco’s Modified Eagle Medium (DMEM), and supplemented with 10% (*v*/*v*) fetal bovine serum (FBS). The cells were maintained at 5% CO_2_, 37 °C, and 95% humidity.

#### 2.3.1. Cell Viability Assay

Different concentrations of Cupuaçu extracts and analytical standards were previously tested to select the proportions that could ensure in vitro cell viability. Pure extracts were tested in dilutions of 10, 50, 80, 100, 150, and 200 µg/mL in DMEM. For this, 1 × 10^4^ cells were plated in 96-well plates and cultured overnight to allow cell attachment into the plates. Then, cells were treated according to the following conditions: untreated (control samples), which were maintained in cell culture media throughout the experiment and treated with vehicle only, being considered as having 100% cell viability; and treated with Cupuaçu extract (samples were maintained in basal media lacking FBS supplemented with the different concentrations of Cupuaçu extract solution in ethanol). After 48 h incubation, the cells were washed with PBS, and fresh DMEM supplemented with FBS was added to each well. At this time, 10 μL of 3-(4,5-Dimethyl-2-thiazolyl)-2,5-diphenyl-2H-tetrazolium bromide (MTT; Sigma-Aldrich, St. Louis, MO, USA) were also added (final concentration of 5 mg/mL), followed by 4 h plate incubation in the dark. Then, the MTT solution was removed, and DMSO was added to dissolve the formazan crystals formed. A microplate spectrophotometer (BioTek, Winooski, VT, USA) was used to determine the absorbance at 570 nm. A minimum of three independent experiments was executed.

#### 2.3.2. Proliferation Assay

The MTT assay was also executed to assess cellular proliferation, using the highest non-toxic concentration of Cupuaçu extract (100 μg/mL) and longer incubation time-points. Unlike in the cell viability assay, fibroblasts were trypsinized and harvested, and 1 × 10^4^ cells were cultured in 96-well microplates. These were left untreated for 24 h for complete attachment to the culture surface in their respective cell culture media. Afterward, the cells were incubated with Cupuaçu extract for 1, 4, and 7 days. At each point in time, an MTT assay was performed. Untreated fibroblasts were kept in DMEM without FBS supplementation as negative cell proliferation control. A minimum of three independent experiments was executed.

#### 2.3.3. Scratch Assay

The Cupuaçu extract’s effect on the migratory behavior of fibroblasts was analyzed as described in Liang et al.’s protocol [22]. Briefly, cells were plated at a density of 2.5 × 105 cells/mL in 6-well plates. After reaching 100% confluence, the wells were scratched using a 200 μL pipette tip. The cultures were washed to remove cell debris and treated with either basal medium containing growth stimuli (i.e., untreated samples for positive migration control) or 100 μg/mL Cupuaçu extract. FBS was removed to decrease the stimulation of cell proliferation, as it may influence experimental outcomes. The cultures were incubated at 37 °C and photographed immediately after the assay and 24 and 48 h later. Wound closure was evaluated using ImageJ (Wayne Rasband, NIMH, Washington, DC, USA), and cell migration ability was measured as per the action of the compound to promote such effect, considering the number of cells that had migrated into the assayed area. A minimum of three independent experiments was executed.

#### 2.3.4. Quantitative Reverse Transcription-Polymerase Chain Reaction (qRT-PCR)

The sequences of the target genes were obtained from Genbank and exported to Primer Express v.3.3 programs (Applied Biosystems, Thermo Fisher, Waltham, MA, USA). The transcripts assessed for cultured cells were B-cell CLL/lymphoma 2 (BCL-2), elastin, basic fibroblast growth factor (FGF-2), MKI-67 proliferation marker (MKI-67), matrix metalloproteinase-1 (MMP-1), hyaluronic acid synthase-2 (HAS-2), and glyceraldehyde 3-phosphate dehydrogenase (GAPDH). After 48 h of treating the cells for migration assay, the total RNA within each group was isolated using Trizol reagent, following manufacturer instructions. The cells cultured wells had the medium removed, and 1 mL of TrizolTM was added (Thermo Fisher, Waltham, MA, USA). After successive pipetting to promote cell lysis, Trizol was added to 1.5 mL DNA-RNAse-free Eppendorf, and 200 μL of chloroform was added to each tube, which was vigorously stirred. After that, the tubes were incubated at room temperature for 2–3 min and centrifuged for 15 min at 4 °C and 13,000 rpm. The upper (aqueous) phase was collected, and the RNA precipitated for 10 min with the addition of isopropyl alcohol. Then, another centrifugation was performed at 4 °C and 13,000 rpm for 10 additional min and washed with 75% ethanol, followed by another 10-min centrifugation at 4 °C and 7500 rpm. The RNA was diluted in autoclaved ultrapure water. RNA concentration was determined by measuring its absorbance at 260/280 nm in a Nanodrop device (NanoDrop, Thermo Fisher, Waltham, MA, USA). RNA samples were reverse-transcribed using the High-Capacity cDNA Reverse Transcription Kit, according to the manufacturer’s instructions. In summary, reverse transcription of the isolated total RNA was performed using 500 ng of the total RNA from each sample, reverse transcriptase (Superscript III, Invitrogen, Waltham, MA, USA), Oligo dT (12–18) primer (Invitrogen, Carlsbad, CA, USA), and RNAse inhibitors, reaching a final volume of 20 μL. Incubation steps were taken according to the time and temperature recommended by the manufacturer. Amplification by quantitative polymerase chain reaction (qPCR) was performed in duplicates. Reactions were prepared with standardized reagents for real-time PCR (SYBRTM Green Master Mix, Thermo Fisher, USA), using the following primers: GAPDH F-ACATCGCTCAGACACCATG, GAPDH R-TGTAGTTGAGGTCAATGAAGGG; BCL2 F-CAAAGCTGCAGGCTGTTTAAG, BCL2 R-GTCTGTCTGTGTGTGTGATGT; MKI67-AACACCATCAGCAGGGAAAG, MKI67 R-CTGCACTGGAGTTCCCATAAA; MMP1-GAGCTTCCTAGCTGGGATATTG, MMP1 R-ACTGGCCTTTGTCTTCTTTCT; FGF2 F-CAAGGACCCCAAGCGGCTGT, FGF2 R-AGCTTGATGTGAGGGTCGCTCTT; ELN F-AAGGCTGCCAAGTACGGAGT, ELN R-CAAACTGGGCGGCTTTGG; HAS2 F-CTCGCAACACGTAACGCAAT, HAS2 R-CAGTGCTCTGAAGGCTGTGT. Amplifications were performed by StepOne Plus equipment (Applied Biosystems, Waltham, MA, USA), and the data were analyzed by StepOne Software v2.3, Foster City, CA, USA [23].

### 2.4. Nanocapsule Preparation and Characterization

The polymeric lipid-core nanocapsules were prepared by the interfacial deposition method of preformed polymers, as previously described by Bender et al. [24]. Briefly, an organic phase containing polycaprolactone (0.1 g), sorbitan monostearate (0.04 g), and caprylic/capric triglyceride (0.12 g) dissolved in acetone (25 mL) was prepared. In parallel, an ethanolic solution (5 mL) was prepared with lecithin (0.03 g) and poured into the organic phase. Under moderate magnetic stirring at 40 °C, this mixture was then poured into an aqueous solution (50 mL) containing polysorbate 80 (0.08 g). After 10 min, the final mixture was evaporated under reduced pressure to eliminate the organic solvents and concentrate the final colloidal solution.

For the coating procedure, 10 mL of a 0.3% (*w*/*v*) chitosan solution in 1% acetic acid was added dropwise under moderate magnetic stirring over the colloidal solution. The reaction medium continued to be stirred for 4 h at room temperature. In this way, two types of nanoparticles were produced: (i) polymeric nanocapsules containing the Cupuaçu extract (NC) and (ii) polymeric nanocapsules containing the Cupuaçu extract coated with chitosan (NC-CH).

The nanocapsules’ hydrodynamic diameter, polydispersity index (PdI), and zeta potential were evaluated using Zetasizer Nano equipment (NANO ZS90, Malvern Instruments, Worcestershire, UK). Before being analyzed, the samples were 1:10 (*v*/*v*) diluted in water. The pH of the nanocapsule suspensions was measured by a calibrated potentiometer (MPA-210 Model, MS-Tecnopon, São Paulo, Brazil) with the direct immersion of the electrode in the nanoformulation. The entrapment efficiency EE (%) of the biomarker methyl oleate in the nanoparticles was determined by separating the free drug (FD) from the nanoparticles’ dispersions. Thus, 1 mL of each sample was centrifuged for 30 min at 2150× *g* using Vivaspin to increase the samples’ concentration. The filtrate was collected and analyzed by GC-MS, according to the method described in Section 2.2. EE (%) was then calculated, taking into consideration the total concentration of methyl oleate used to prepare each nanoparticle sample (TD), according to the following equation:

EE% = [(TD − FD)/TD] × 100(1)

The morphology of polymeric nanocapsules was analyzed by transmission electron microscopy (TEM, model JEM 1011, Tokyo, Japan). The samples were diluted in a ratio of 1:200 (*v*/*v*), placed in gold gratings to be covered with “formvar” resin, and left drying at room temperature. Next, the excess formulation was removed with filter paper. After this step, 3 μL of 3% (*w*/*v*) uranyl acetate solution was added and allowed to dry, protected from light for 5 additional min at room temperature. Again, the excess solution was removed with filter paper, and the sample was analyzed in the equipment.

### 2.5. Mucoadhesion Assay

Mucoadhesiveness of chitosan nanoparticles was evaluated in vitro as previously described [18]. Porcine mucin (type II) was hydrated overnight in ultrapure water at 4 °C, with a ratio of 1:10 (*w*/*v*). The pH was then adjusted to 7.4 with 1.0 mol/L NaOH. The solution was diluted in HEPES buffer (pH 7.4) with a final mucin concentration equal to 1% (*w*/*v*) and was sonicated with a tip sonicator (Sonics, Newtown, CT, USA) to produce mucin nanoparticles with a hydrodynamic diameter smaller than 500 nm. Mixtures of the NC and NC-CH with mucin nanoparticles were prepared, mixing equal volumes of colloidal dispersions and homogenizing (Vortex-Genie 2TM Daigger, Bohemia, NY, USA) for different periods (0, 3, 12, 15, 24, and 30 min). As described above, nanoparticle dispersions hydrodynamic diameter and zeta potential were measured before and after the mixture with mucin. The section size statistics were calculated in triplicates using the Zetasizer Nano Series device.

### 2.6. Stability

The physical stability of the nanocapsules was determined at 0, 7, 15, 30, 70, and 120 days after their production, in which samples of NC and NC-CH (*n* = 3) were stored at temperatures of 8 °C ± 3.0 and 25 °C ± 2.0 and possible changes in the average hydrodynamic diameter, PdI, zeta potential, and pH were monitored.

### 2.7. Irritation Assay

Skin irritation was also evaluated using reconstructed human epidermis (RHE) provided by EpiSkin™, according to [25] OECD guidelines described as test *n*. 439 in 24-well plates. The samples tested in this study were NC, NC-CH, potentially irritating positive control (5% sodium dodecyl sulfate solution, SDS 5%), and potentially non-irritating negative control (0.9% sodium chloride solution, NaCl 0.9%) (*n* = 3). Cell viability was measured by MTT (1 mg/mL) reduction in a microplate spectrophotometer at 570 nm (Bio-Tek PowerWave model HT, Winooski, VT, USA). For this protocol, a substance was determined as irritating when the percentage tissue viability—assessed by MTT assay—was less than or equal to 50% and as non-irritating when this percentage was above 50% [26].

### 2.8. Skin Penetration

In vitro penetration was evaluated in intact and burnt porcine skin samples following a modified Saarbrücken method [27]. Briefly, quintuplicates of previously prepared porcine skin samples were used (5.7-cm^2^). The burnt skin sample simply consisted of porcine skin that had been burnt for 7 sec by a hot plate at 85 °C [28]. Permeation was carried out for 24 h by adding 2 mL of NC-CH and NC onto the skin surface. At the experiment’s conclusion, the skin surface was cleaned with humidified cotton, and the skin was further cut into small pieces with a scissor. The fragments were placed in glass containers with 5 mL of hexane and were left unstirred overnight. The resulting aliquots were filtrated in 0.45 μm pore filters, and the filtrate was analyzed by GC-MS (Section 2.10).

### 2.9. Biomarker Quantification

Oleic acid was chosen as a biomarker of the extract for skin penetration studies. For this, samples from the permeation studies were previously submitted to the esterification process described by Bannon et al. [29] adapted for the analysis. Hence, samples were analyzed as per its methyl oleate content. The quantification followed the same analytical procedures employed during extract characterization (Section 2.2). This method was selective and linear (concentration ranging from 10–50 μg/mL, r = 0.999), following the International Conference on Harmonization (ICH) guidelines. The limit of detection (3.086 µg/mL) and limit of quantification (9.351 µg/mL) proved to be efficient in quantifying the natural extract marker obtained in the extraction process (99.77–100.97%).

### 2.10. Data Analysis

All experiments were performed as technical and biological triplicates. Permeation experiments were performed in quintuplicates. Data were analyzed considering the analysis of variance and using GraphPad Prism^®^ Software, version 7.02 (San Diego, CA, USA). The significance level was set at *p* < 0.05.

## 3. Results and Discussion

### 3.1. Characterization of Cupuaçu Seed Extracts

Oleic acid was identified as the principal constituent of the Cupuaçu seed extract, comprising 41.81% of the extracted content. The second main constituent is stearic acid (35.79%), followed by palmitic acid (8.67%), as shown in Table 1. The chemical composition of hexane extract is according to previous studies, which described ranges of 39–47%, 22–35%, and 6–12% to oleic, stearic, and palmitic acids, respectively [30,31]. These results justify oleic acid (methyl oleate) as a biomarker for GC-MS quantification analyses in skin penetration studies.

### 3.2. In Vitro Biological Activity

#### 3.2.1. Cell Viability and Proliferation

**Figure 1:**
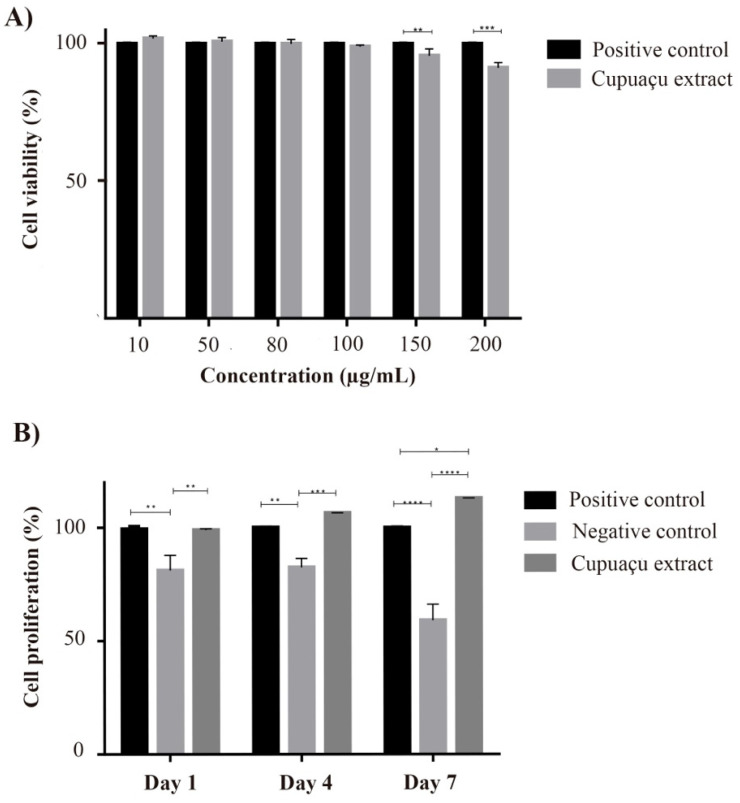
Primary human dermal fibroblast response to Cupuaçu extract treatment. (**A**) Cellular viability after 24 h incubation with different concentrations of Cupuaçu extract; (**B**) cellular proliferation for up to 7 days incubation with 100 μg/mL Cupuaçu extract. The graphs represent the absorbance mean value and standard deviation of three biological replicates in triplicates, normalized according to the 100% viability/proliferation positive controls. Positive controls received FBS supplementation and negative controls consisted of cells maintained without FBS supplementation. Statistical differences have been verified by two-way ANOVA test and Tukey post hoc test. * *p* < 0.05; ** *p* < 0.01; *** *p* < 0.001; **** *p* < 0.0001.

The skin wound healing process includes an inflammatory response, the formation of granulation tissue, and matrix remodeling. In this context, several different cellular processes must occur in a controlled manner to guarantee optimal wound healing. Among such processes, cellular proliferation, migration, and contractility have been pointed out as critical events for regeneration, cell proliferation and migration necessary to populate the wound bed, and cell contractility contributing to wound closure [32]. Fibroblasts comprise the main cell type of connective tissue and play essential roles in the regeneration of different anatomical sites, including the skin, proliferating to replace lost cells, producing reparative growth factors, and depositing extracellular matrix components to support further cellular migration and future tissue function [33]. Fatty acids can be valuable actives for the wound healing process because of their metabolic actions. The obtained data indicated that the fibroblasts maintained their viability after 24 h of incubation with Cupuaçu seed extract (Figure 1A). Thus, 100 µg/mL concentration was selected to be used in further experiments, as this was the highest concentration that did not affect cell viability. Not only did the Cupuaçu extract maintain fibroblast viability, but it also induced cellular proliferation in all-time points assessed (Figure 1B). After 4 and 7 days of treatment, such proliferation was significantly higher than positive (FBS) and negative (no FBS) controls (*p* < 0.05). To the best of our knowledge, such biotechnological potential of the frequently discarded Cupuaçu seed is demonstrated here for the first time, aggregating value to its production chain.

#### 3.2.2. Cell Migration

**Figure 2:**
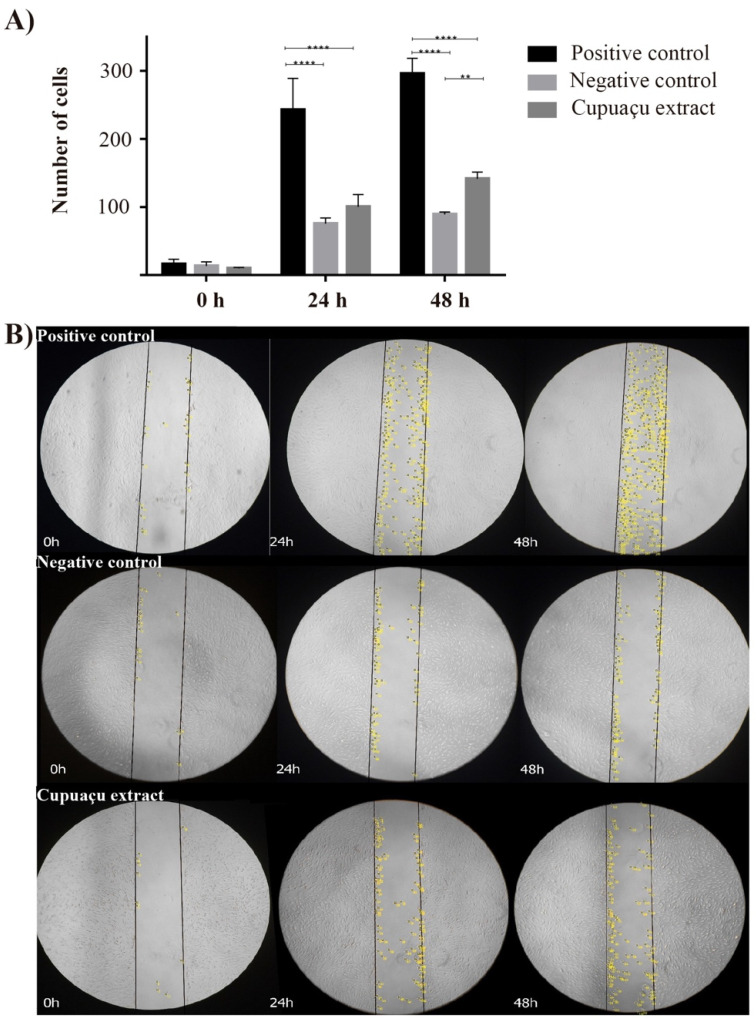
Migratory behavior of fibroblasts treated with 100 μg/mL Cupuaçu seed extract. (**A**) cellular migration normalized to the positive controls in each time point. (**B**) Wound closure immediately after scratch, 24 and 48h later. Positive controls received FBS supplementation and negative controls consisted of cells maintained without FBS supplementation. Statistical differences have been verified by two-way ANOVA test and Tukey post hoc test. ** *p* < 0.01; **** *p* < 0.0001. Yellow dots represent a migrating cell at that specific time.

Cell proliferation and cell migration are relevant events for the tissue regeneration process, especially in the context of extensive wound healing, in which it is imperative to prevent water loss and tissue infection. Currently, most treatment regimens in this context focus on preventing infection and protecting the lesion bed with dressings containing expensive cell proliferation and migration-inducing recombinant factors, such as PDGF (e.g., Regranex^®^). As depicted in Figure 2, the Cupuaçu seed extract treatment induced a significantly higher cell migration of treated samples than the negative control at 48 h (*p* < 0.05). Therefore, it is possible to suggest that the Cupuaçu extract holds promising skin regenerative potential for treating different skin lesions, including burns.

#### 3.2.3. Gene Expression

**Figure 3:**
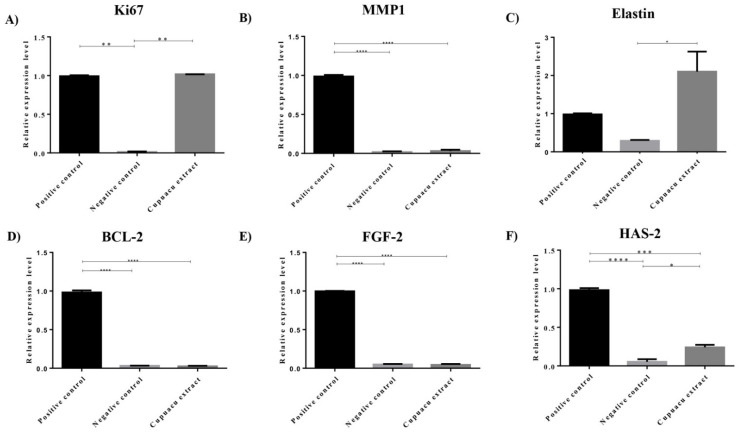
Effect of the Cupuaçu seed extract on the mRNA levels of (**A**) Ki-67, (**B**) MMP-1 (matrix metalloproteinase-1), (**C**) Elastin, (**D**) BCL-2, (**E**) FGF-2 (fibroblast growth factor-2), and (**F**) HAS-2 (hyaluronic acid synthase-2) genes. Positive control represents cells maintained in basal medium with FBS, and negative control maintained in the absence of FBS. Statistical differences were verified by two-way ANOVA test and Tukey post hoc test. * *p* < 0.05; ** *p* < 0.01; *** *p* < 0.001, **** *p* < 0.0001.

Despite the complexity of the tissue regeneration process, different genes have been investigated and serve as relevant parameters to establish the regenerative potential of new experimental regenerative treatments [34]. Here, we decided to investigate the mRNA expression of MKI67, a cellular proliferation marker, BCL-2, an apoptosis regulating gene, in addition to FGF2, which is a relevant growth factor for fibroblast signaling, in addition to the ECM remodeling genes MMP1, elastin (ELN) and HAS2 (Figure 3).

Experimental results showed that, after 48 h treatment of dermal fibroblasts with Cupuaçu extract, it was possible to observe a significant induction of mRNA expression of MKI67, Elastin, and HAS2 (*p* < 0.05, compared to the negative control), which corroborate the observations made in the proliferation assay and support the notion that Cupuaçu extract might contribute to the reestablishment of a functional skin ECM that supports skin elasticity [35] and hydration post-lesion, without promoting excessive HAS2 expression, which has been associated with abnormal keratinocyte migration [36].

According to the literature, while cellular proliferation is a relevant event in the proliferative phase of wound healing, tissue continuity is reestablished. Tissue maturation follows and depends on optimal ECM deposition and a delicate balance in cell signaling to prevent fibrotic scarring. In this sense, FGF2 has been already described as both fibrotic [37,38] and anti-fibrotic [39], the fact that Cupuaçu extract does not interfere with FGF2 gene expression under the absence of other stimuli possibly being interpreted as a neutral, positive effect. Similarly, despite the relevance of MMPs in the scarless tissue regeneration observed in fetal compared to adult skin [40], the excessive MMP expression has also been correlated with poor lesion healing [41]. Once again, the lack of MMP1 induction by Cupuaçu extract treatment may also be considered compatible with a reparative gene expression profile. Finally, the absence of BCL2 mRNA induction by Cupuaçu extract may corroborate the absence of cellular toxicity exerted by the extract in the tested concentration. Therefore, taken together, the functional and molecular tests presented are coherent and support the notion that the Cupuaçu extract may hold a positive impact on skin wound healing.

### 3.3. Nanocapsules Production and Characterization

**Figure 4:**
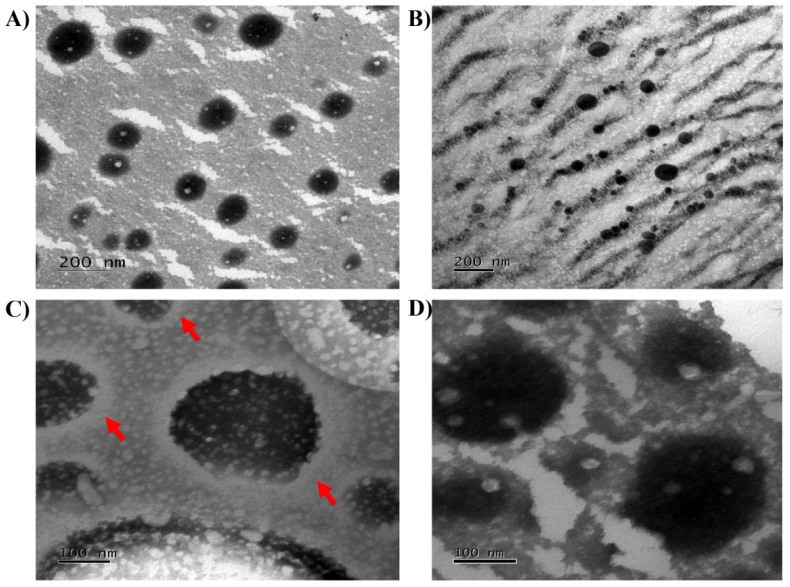
Photomicrographs obtained by TEM. (**A**) Blanc NC (20 K); (**B**) Blanc NC-CH (20 K), (**C**) NC-CH (15 K) and (**D**) NC (15 K). Red arrows represent a “white halo” around the chitosan-coated nanocapsules.

The biotechnological potential of Cupuaçu extract being established, the next challenge is successfully delivering such a biological product into the skin. For this, polymeric nanocapsules incorporating the Cupuaçu seed extract were successfully produced. Table 2 shows the average size, PdI, zeta potential, pH, and loading efficiency of both polymeric nanocapsules containing the Cupuaçu extract (NC) and polymeric nanocapsules containing the Cupuaçu extract coated with Chitosan (NC-CH). NC had negative characteristics and, after coating and as expected, their zeta potential became positive. Independent of chitosan coating, EE% was extremely high (>94%), which was expected for this type of nanosystem and such a lipophilic extract. The chitosan coating did not alter particle morphology (Figure 4). Both NC and NC-CH were spherical and formed a relatively monodisperse, unimodal population of nanocapsules.

### 3.4. Mucoadhesive Assay

Chitosan has been extensively investigated in developing controlled drug delivery systems for a wide range of drug delivery routes [13,18,42], mainly due to its mucoadhesive properties that facilitate formulation-tissue interaction and, consequently, drug absorption [43].

**Figure 5:**
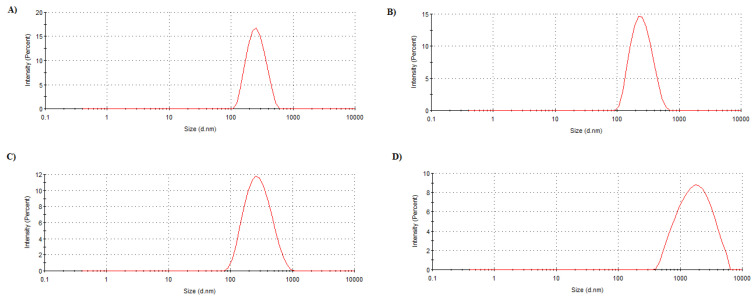
In vitro evaluation of NC and NC-CH mucoadhesiveness. Distribution of hydrodynamic diameter for (**A**) NC; (**B**) NC mixed with mucin nanoparticles; (**C**) NC-CH; (**D**) NC-CH mixed with mucin nanoparticles.

The in vitro mucoadhesion test confirmed that the chitosan coating confers this characteristic to the nanocapsules (Figure 5). Only after NC-CH was mixed with NP-MCN (358.4 ± 27.08 nm) was there an increase in the average diameter of the nanosystem population (1203.2 ± 258.79 compared to 229.3 ± 0.10 after mixture with uncoated nanocapsules).

### 3.5. Stability

**Figure 6:**
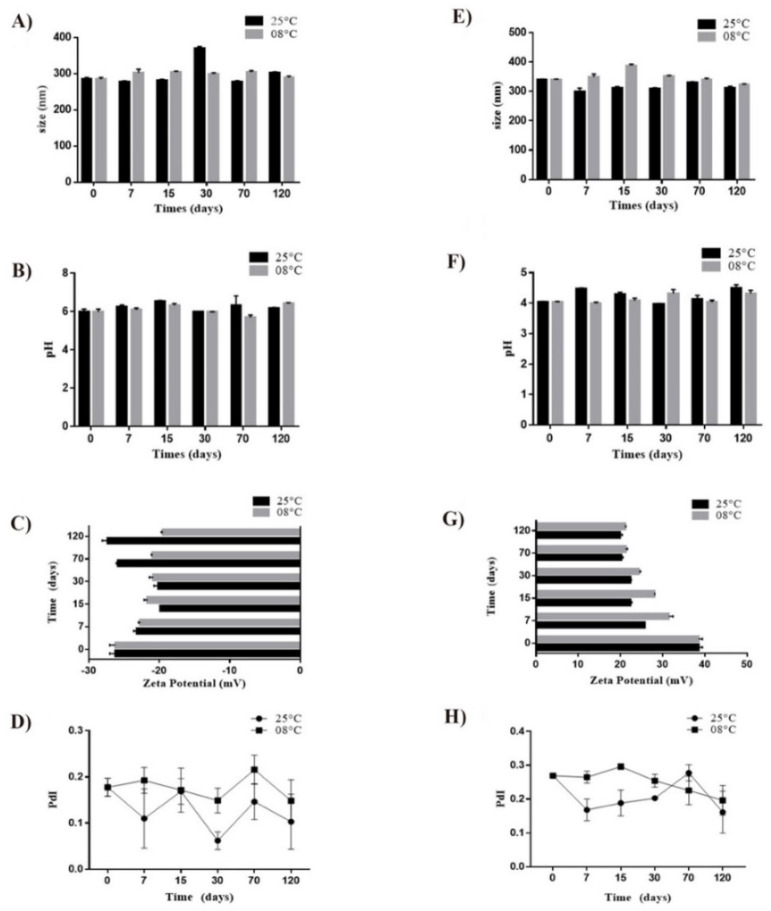
Evaluation of average size, pH, zeta potential, and PdI for NC (**A**–**D**) and NC-CH (**E**–**H**) stored at 8 ± 3.0 °C and 25 ± 2.0 °C, over 0, 7, 15, 30, 70, and 120 days.

Nanocapsules showed to be stable for up to 120 days of storage (Figure 6). Nanocapsule hydrodynamic diameter exhibited only minor oscillations, and, in general, nanocapsules preserved their monodispersity characteristics based on the PdI values, which were maintained below 0.3. The NC maintained an average size of approximately 300 nm, while NC-CH indicated a size between 300 and 400 nm. The zeta potential was above ±30 mV. The pH was maintained around 4.0–4.5 for NC-CH and 5.0–6.0 for NC. Notably, there were no differences from the samples stored at 8 or 25 °C, which indicates that there might not be a need to maintain this formulation refrigerated.

### 3.6. Irritation Assay

**Figure 7:**
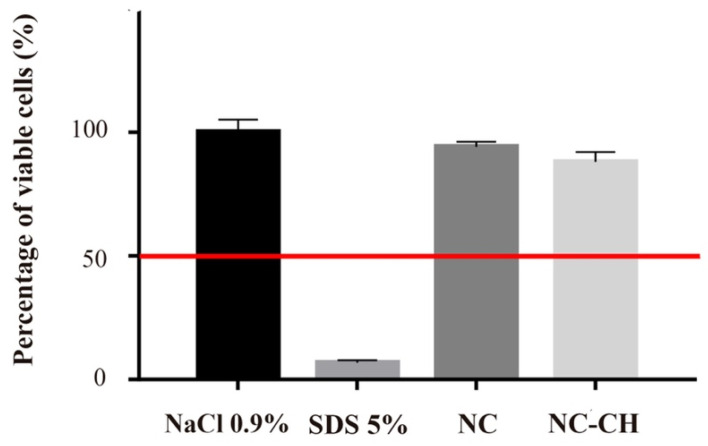
Cell viability percentage after the exposure of Reconstructed Human Epidermis (RHE) to either NC or NC-CH. Positive and negative controls comprised SDS 5% and NaCl 0.9% solutions, respectively. The data represent the means of 3 replicates ± standard deviation.

Skin irritation is defined as reversible damage produced after exposing the skin to irritating materials. The clinical manifestation of irritation is characterized by erythema and edema resulting from a series of cascading events initiated by the penetration of the irritating chemical agent into the stratum corneum. Despite the absence of cellular toxicity observed in human dermal fibroblast cultures, we assessed whether the final formulation carrying the Cupuaçu extract would present any irritation potential, using the EpiSkin™ RHE model, which assesses the irritation potential of chemical agents through the initial events of the inflammatory cascade by measuring cell viability (OECD, 2010). Figure 7 represents the cell viability percentage. The positive control (SDS 5%) showed cell viability of 6.75 ± 1%. In comparison, the cell viability percentage for the negative control (NaCl 0.9%) was 100 ± 5%. In turn, NC resulted in 94.20 ± 2%, and NC-CH in 88.04 ± 4%. Based on OECD criteria, the two nanoformulations presented results close to the negative control and were therefore classified as non-irritating and had their safety ensured for topical application.

### 3.7. Skin Penetration

**Figure 8:**
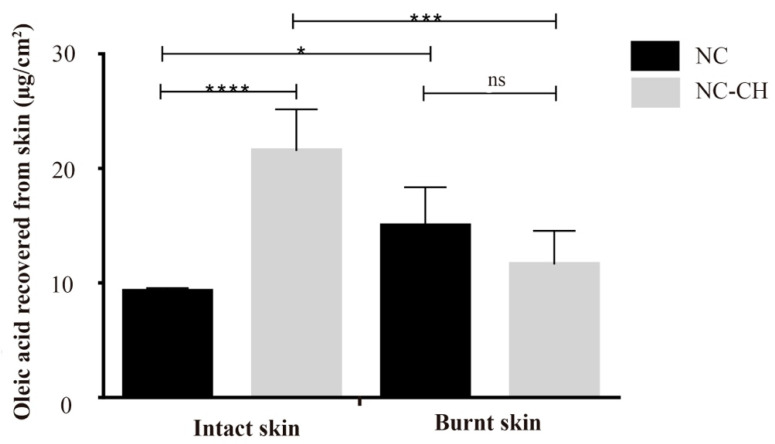
Penetration of oleic acid in burnt and intact skin samples treated 24 h with NC or NC-CH. *ns* non significant; * *p* < 0.05; *** *p* < 0.001, **** *p* < 0.0001.

Although the wound healing potential of the Cupuaçu seed extract could be demonstrated in cell culture experiments, the actual wound healing therapeutic potential can only be fully explored if the phytotherapeutic can reach the target cells from a safe topical formulation. Therefore, when evaluating the skin penetration of the molecules of interest, considering their purpose, i.e., to promote wound healing to damaged skin tissue, the state of the skin is a crucial factor. For this, the penetration of the Cupuaçu biomarker (oleic acid, the main constituent, 41.81%) was evaluated in two skin models, intact and burnt skin (Figure 8).

Results demonstrated, as expected, that the chitosan coating almost doubled the oleic acid penetration into the intact skin (21.5 ± 3.6 μg/cm^2^) (*p* < 0.05) when compared to NC (9.3 ± 0.3 μg/cm^2^). However, such a penetration enhancing effect is not observed in burnt skin samples, in which there were no differences in penetration amount (~15.0 μg/cm^2^) despite the coating (*p* > 0.05). These are entirely plausible results when considering the mechanism by which chitosan promotes skin penetration, i.e., chitosan acts by transiently widening the tight junctions between cells [20]. Together with the lipid matrix, these cellular junctions and scaffolding proteins act as important barrier-function structures within the innermost layer of the stratum corneum, the stratum conjunctum. Therefore, in the burnt skin model applied in these experiments, the stratum corneum had been wholly removed; such an effect is absent. Nevertheless, the skin might be partially damaged and partially intact or even contain part of the stratum corneum in an actual physiological situation. In such cases, chitosan-coated nanocapsules may represent a superior delivery platform. Additionally, as previously described, chitosan presents well-described relevant properties to wound healing [44,45]. Besides being biodegradable and biocompatible, chitosan presents a moderate antimicrobial activity and positively influences the re-epithelialization and regeneration of the granular layer of wounds [17]. Hence, a beneficial effect might also be expected for the wound regions uncovered by the stratum corneum. Such formulations, allied with the biotechnological potential demonstrated for the Cupuaçu seed extract, might be promising for wound healing in general. Previous works in the literature have evaluated different polymer matrices [46] and even Cupuaçu pulp extract for the obtention of Kefir Biofilms [47] that could be used as natural healing biofilms. However, to the best of our knowledge, this is the first time seed extract biotechnological potential is explored.

## 4. Conclusions

In the present study, we have demonstrated, for the first time, the potential biotechnological value of Cupuaçu almonds, which are often discarded as a food industry waste, as a promising pharmaceutical ingredient for the development of skin regenerative products. Cupuaçu seed extract showed to be non-toxic and to induce cell proliferation, migration, and a reparative gene expression profile in primary human dermal fibroblast cultures. Furthermore, when incorporated in a nanoparticulated system, the extract was non-irritant. Additionally, chitosan coating the nanocapsules promotes active penetration into undamaged skin areas, which are still covered by the stratum corneum. Hence, chitosan-coated nanocapsules loaded with Cupuaçu seed extract represent promising delivery systems for skin regeneration.

## Figures and Tables

**Table 1:** Chemical composition of the Cupuaçu seed extract in percentage determined by CGMS.

Compounds	Percentage in Extract (%)
Oleic acid	41.81
Stearic acid	35.79
Palmitic acid	8.67
Eicosanoic acid	7.85
Linoleic acid	2.94
Behenic acid	1.09
Methyl (11E)-11-icosenoate	0.38
Ethyl oleate	0.28
Myristic acid	0.15
Not identified	0.48
Total	99.44

**Table 2:** Characterization of chitosan-coated (NC-CH) and uncoated nanocapsules (NC), including hydrodynamic diameter (nm), polydispersity index (PDI), zeta potential (mV), entrapment efficiency (%), and pH (*n* = 3).

Sample	Hydrodynamic Diameter (nm)	PDI	Zeta Potential (mV)	EE%	pH
NC	278.3 ± 5.0	0.18 ± 0.02	−26.2 ± 0.8	94.6 ± 0.7	5.5
NC-CH	337.2 ± 2.1	0.27 ± 0.01	+ 38.5 ± 0.9	94.4 ± 0.6	4.5
